# Comprehensive circRNA expression profile during ischemic postconditioning attenuating hepatic ischemia/reperfusion injury

**DOI:** 10.1038/s41598-018-36443-8

**Published:** 2019-01-22

**Authors:** Pengpeng Zhang, Yingzi Ming, Qifa Ye, Ying Niu

**Affiliations:** 1 Department of Transplant Surgery, The Third Xiangya Hospital, Central South University, Changsha, 410013 China; 2 Zhongnan Hospital of Wuhan University, Institute of Hepatobiliary Diseases of Wuhan University, Transplant Center of Wuhan University, Hubei Key Laboratory of Medical Technology on Transplantation, Wuhan, Hubei 430071 China

## Abstract

Ischemic postconditioning (IPO) attenuates hepatic ischemia/reperfusion (I/R) injury. The aim of this study was to explore the role of circular RNAs (circRNAs) in the protective mechanism of IPO. In this study, microarray hybridization analysis was performed to determine the circRNA expression profile. Briefly, a total of 1599 dysregulated circRNAs were detected. The competitive endogenous RNA (ceRNA) network, including 6 circRNAs, 47 miRNAs and 90 mRNAs, indicated that the potential “housekeeping” function of circRNAs is dysregulated in hepatic I/R injury. Based on the validation results of selected circRNAs, miRNAs and mRNAs following qRT-PCR amplification, the mmu_circRNA_005186-miR-124-3p-Epha2 pathway was constructed. Dual-luciferase reporter analysis showed that miR-124-3p interacted directly with mmu_circRNA_005186 and Epha2 through the predicted binding sites, which suggested that mmu_circRNA_005186, serving as a miRNA sponge for miR-124-3p, regulated the expression of Epha2. Functionally, we explored the mechanism of mmu_circRNA_005186 in LPS-treated RAW264.7 cells which simulated the inflammation in hepatic I/R injury. We found that mmu_circRNA_005186 silencing attenuated the LPS-induced inflammation and was associated with miR-124-3p upregulation and Epha2 downregulation. Our study is the first to show that circRNAs are closely related to hepatic I/R injury and IPO and suggests that targeting mmu_circRNA_005186-miR-124-3p-Epha2 pathway might attenuate hepatic I/R injury.

## Introduction

Circular RNAs (circRNAs), a novel type of endogenous noncoding RNA that was recently rediscovered, develop covalently closed loop structures without 5′–3′ polarities^1^. Unlike the structure of linear RNA, which is terminated with a 5′ cap and 3′ tail, ‘backsplicing’ in which an upstream splice acceptor combines with a downstream splice donor, is the main characteristic of circRNA formation^2^. This special structure confers resistance against RNAase, making circRNA more stable than linear RNA and more ubiquitously expressed in tissues^1^. Although the function of circRNA in animals is still uncertain, the “miRNA sponge” trait of circRNA has become a popular research topic. The expression of downstream mRNA decreases when circRNA binds to its target miRNA, acting as a sponge to inhibit transcription, further influencing the pathophysiological processes of various diseases^3,4^.

Hepatic ischemia/reperfusion (I/R) injury is an inevitable and serious problem that occurs in clinical post-operative settings, such as after liver transplant or partial hepatectomy, that leads to pathological cellular changes and organ failure^5^. Jaeschke *et al*. verified that two obvious phases occur in acute liver injury after hepatic I/R^6^ and that Kupffer cells (KCs), the resident macrophages of the liver, play a pivotal role in the pathogenesis of I/R-induced acute liver injury^7^. Once KCs are activated, proinflammatory cytokines are produced and released, such as tumor necrosis factor α (TNF-α) and interleukin1β (IL-1β), which subsequently activates KCs to amplify the inflammatory loop and promotes neutrophil infiltration into the hepatic microcirculation^8^.

Currently, several mechanical and pharmacological methods have been identified that attenuate liver I/R in animal studies, and ischemic postconditioning, composed of several brief cycles of ischemia and reperfusion before the reperfusion phase, has been used to attenuate I/R injury in organs including the heart^9^, bowel^10^, kidney^11^, brain^12^ and liver^13^. Multiple studies have found that minimizing cell necrosis and apoptosis is crucial for the benefits of IPO^14^. However, no studies have reported circRNA expression profiles in hepatic I/R injury, and whether IPO can attenuate liver I/R injury by mediating circRNA expression is unknown. Therefore, in this study, considering the significance of hepatic I/R injury, we explored circRNA expression patterns via microarray hybridization and predicted the functions of the host genes of differentially expressed (DE) circRNAs. We identified an important role of circRNAs in ischemic postconditioning attenuating hepatic I/R injury.

## Results

### IPO attenuated liver I/R injury

**Figure 1:**
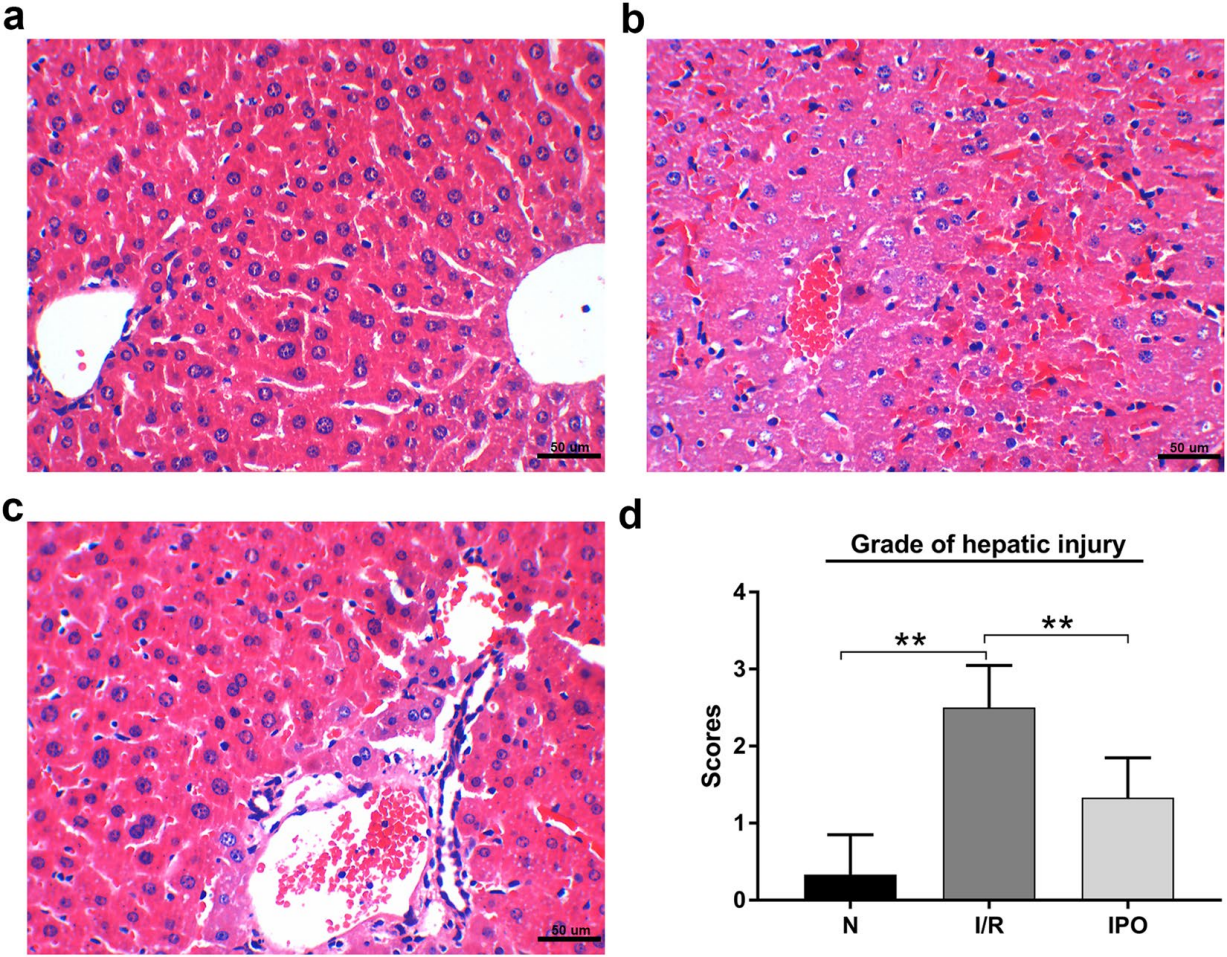
Representative images of hematoxylin and eosin (H&E) staining (magnification 400X, Scale Bar = 50 um). (**a**) Normal liver tissue and **(b)** hepatic ischemia-reperfusion injury liver tissue. **(c)** IPO improved histological injury. **(d)** Histological scores and hepatic injury grade. *p < 0.05, **p < 0.01.

To determine whether IPO can attenuate hepatic I/R injury in this animal model, H&E staining was used to evaluate the histological injury grade. The H&E staining results showed that IPO alleviated the histological injury severity of the hepatic tissue (Fig. 1).

### CircRNA expression pattern during hepatic I/R injury assessed via microarray

**Figure 2:**
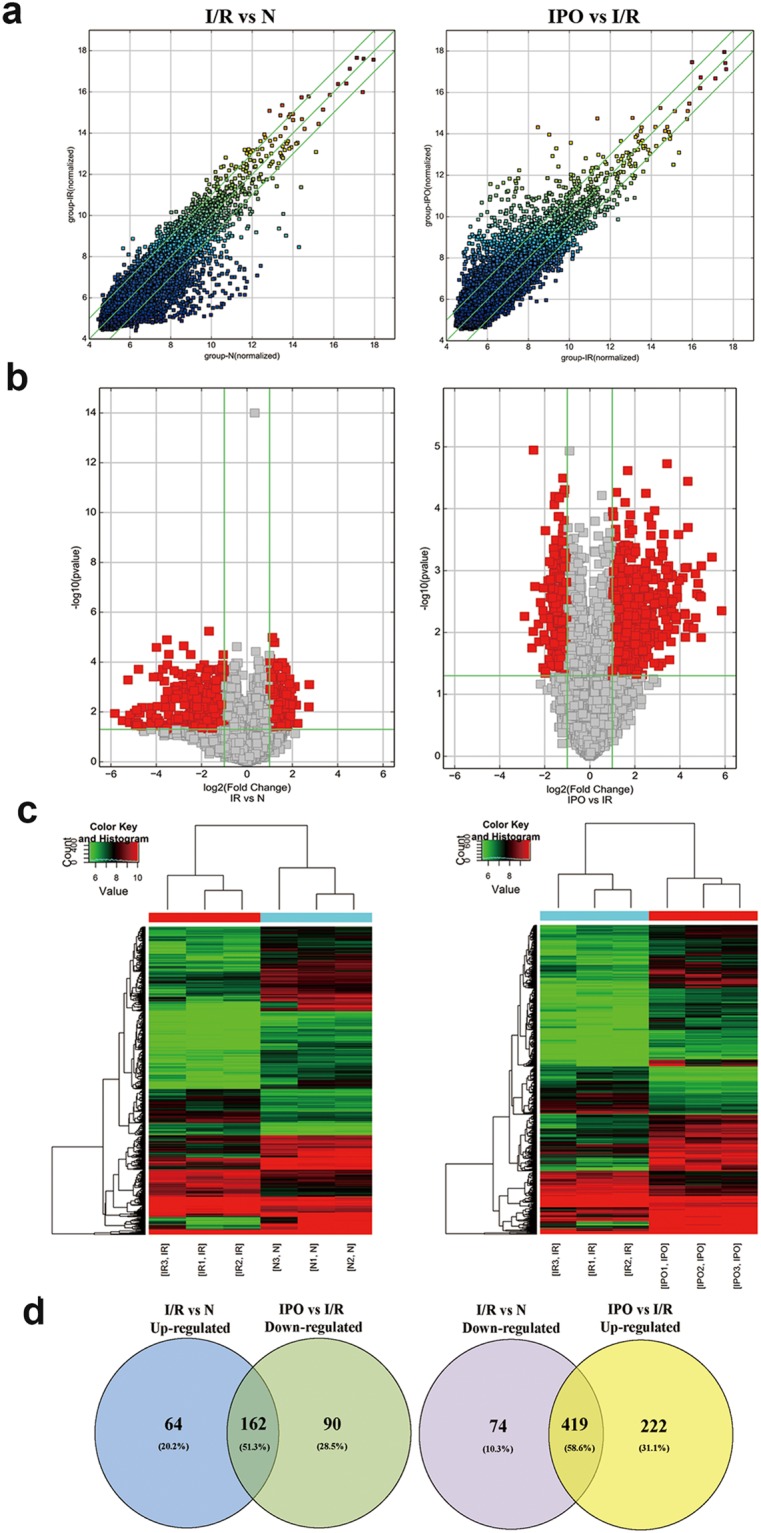
Bioinformatic analysis of circRNA expression pattern during hepatic I/R injury by microarray. (**a**) Scatter plot analysis was conducted to exhibit the circRNA expression distribution. The green lines represent the default significant fold change (2.0). **(b)** A volcano graph was created to show significantly dysregulated circRNAs (fold change >2.0, *p*-value < 0.05). **(c)** Heat map evaluation of the distinguishable circRNA expression patterns among the normal, I/R, and IPO group samples. Each column represents the expression pattern of one sample, and high and low expression levels are indicated by the “red” and “green” lines, respectively. **(d)** Venn diagram analysis of dysregulated circRNAs indicated that most of them (51.3% and 58.6%) were sequentially expressed during IPO attenuation of I/R injury.

Microarray hybridization is an efficient method for detecting differential expression profiles and biological functions of circRNAs. The results showed that a total of 1599 circRNAs were differentially expressed (*p* < 0.05 and fold change ≥2.0). Of these, 213 circRNAs were upregulated and 493 were down-regulated in the I/R group compared with the normal group. At the same time, 641 and 252 circRNAs were up-regulated and down-regulated, respectively, between the IPO and I/R groups (Fig. 2a,b). Hierarchical clustering analysis (heat map) was used to evaluate significantly expressed circRNAs, which were indicated by *p* < 0.05 and a fold change ≥2.0, among the normal, I/R and IPO groups (Fig. 2c). A Venn diagram analysis of the dysregulated circRNAs indicated that most of these RNAs (51.3% and 58.6%) were sequentially expressed (up-regulated in the I/R group and down-regulated in the IPO group) during IPO-attenuated I/R injury (Fig. 2d).

### The predicted biological function of circRNA linear transcripts

**Figure 3:**
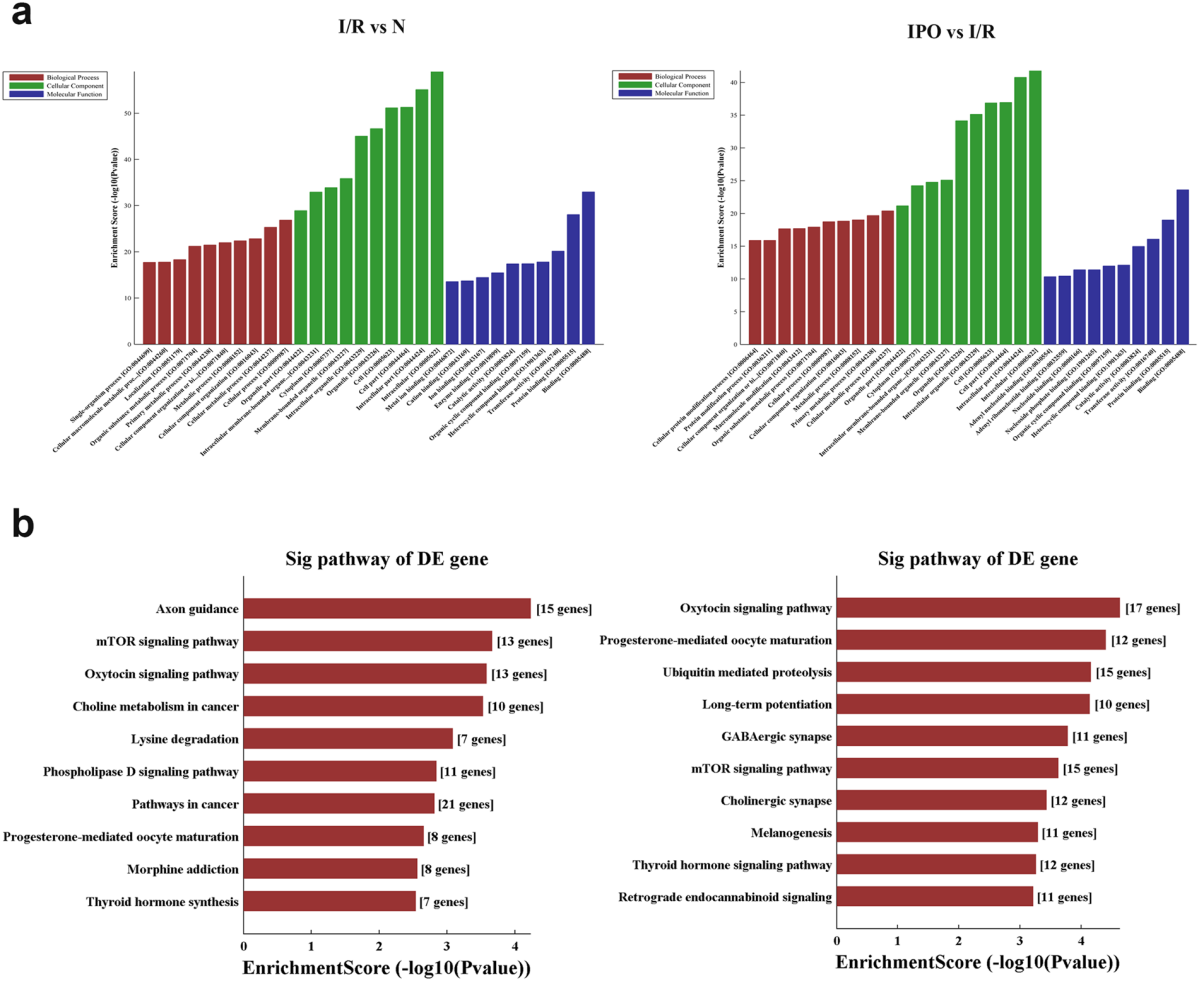
GO and KEGG pathway analysis of dysregulated circRNA linear transcripts. (**a**) GO annotation of the I/R vs N and IPO vs I/R groups via the top 10 enrichment scores in the BP, CC and MF domains. **(b)** KEGG pathway analysis of the I/R vs N and IPO vs I/R groups via the top 10 enrichment scores.

To provide a comprehensive understanding of circRNAs, the linear transcripts of the corresponding origination genes for circRNAs were annotated and were further evaluated by Gene Ontology (GO) and Kyoto Encyclopedia of Genes and Genomes (KEGG) pathway analyses. The GO analysis results from the Biological Process (BP), Cellular Component (CC) and Molecular Function (MF) domains are shown. The top 10 dysregulated GO processes in each domain were analyzed and ranked according to *p*-value (*p* < 0.05). The results indicated that 1420 GO terms were dysregulated (*p* < 0.05) between the I/R and normal group and that 1745 terms were dysregulated between the IPO and I/R group. The top 10 GO terms from the BP, CC and MF domains in the comparison group are exhibited according to the enrichment score and are ranked by *p*-value; all of the top 10 GO terms for each domain were nearly identical (Fig. 3a). This result revealed that in the BP domain, the most meaningful, enriched GO terms were cellular metabolic process, primary metabolic process, metabolic process and cellular process. For the CC domain, the most meaningful, enriched GO terms were intracellular, intracellular part, cell part and cell. In addition, in MF the most meaningful, enriched GO terms were binding, protein binding and transferase activity. In addition, KEGG pathway analysis was performed, and the top 10 pathways in the compared groups were listed according to the enrichment score and were ranked by *p*-value (Fig. 3b). The results indicated that in the protective IPO mechanism, the role of circRNA is closely related to the mechanistic target of rapamycin (mTOR) signaling pathway and further validation are required to confirm these results.

### Validation of selected circRNAs during hepatic I/R injury by qRT-PCR

**Figure 4:**
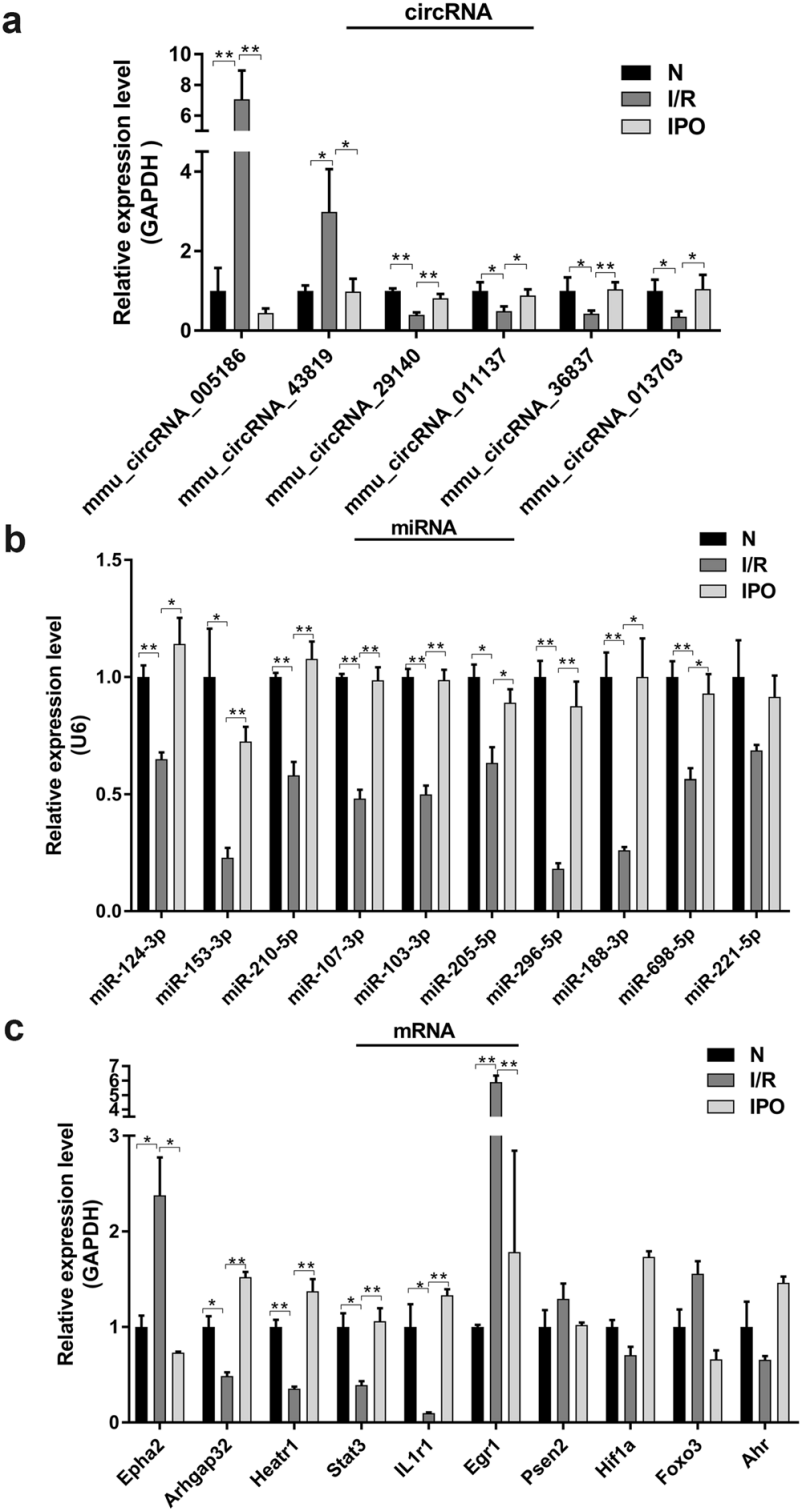
Validation of selected circRNAs, miRNAs and mRNAs by qRT-PCR. (**a**) 6 circRNAs were significantly amplified by qRT-PCR and consistent with the microarray results. **(b)** 9 miRNAs were significantly amplified by qRT-PCR and were inversely correlated with their corresponding circRNAs. **(c)** 6 mRNAs were significantly amplified by qRT-PCR while only Epha2, Arhgap32, Heatr1 and Egr1 had an identical expression trend with their corresponding circRNAs. *p < 0.05, **p < 0.01.

Ten sequentially expressed circRNAs among the normal, I/R and IPO groups were screened according to *p*-value, fold change, raw intensity and type (Table 1) in validation experiments. The results confirmed that consistent with the microarray results, mmu_circRNA_005186, mmu_circRNA_011137, mmu_circRNA_013703, mmu_circRNA_29140, mmu_circRNA_36837, and mmu_circRNA_43819 were significantly amplified by qRT-PCR (Fig. 4a).

**Table 1:** Ten dys-regulated circRNAs were screened for validation by qRT-PCR.

circRNA Name	Type	FC*	Best linear transcripts	Gene Symbol	P-value
**up-regulated (I/R vs Normal)**					
mmu_circRNA_005186	exonic	3.987	NM_010139	Epha2	0.004
mmu_circRNA_44049	exonic	3.175	NM_027889	Vps11	0.007
mmu_circRNA_29064	exonic	3.126	NM_001081254	Fam186b	0.005
mmu_circRNA_32403	exonic	3.052	NM_175459	Glis3	0.003
mmu_circRNA_43819	exonic	2.148	NM_027911	Raver1	0.008
**down-regulated (I/R vs Normal)**					
mmu_circRNA_30491	exonic	15.050	NM_001010833	Mdc1	0.028
mmu_circRNA_013703	exonic	7.142	NM_001024205	Nufip2	0.002
mmu_circRNA_29140	exonic	4.879	NM_009624	Adcy9	0.022
mmu_circRNA_011137	exonic	2.326	NM_177464	R3hcc1l	0.038
mmu_circRNA_36837	exonic	2.138	NM_009649	Akap2	0.019

*FC: Fold Change.

### Construction of the circRNA-miRNA-mRNA network and validation of predicted miRNAs and mRNAs

**Figure 5:**
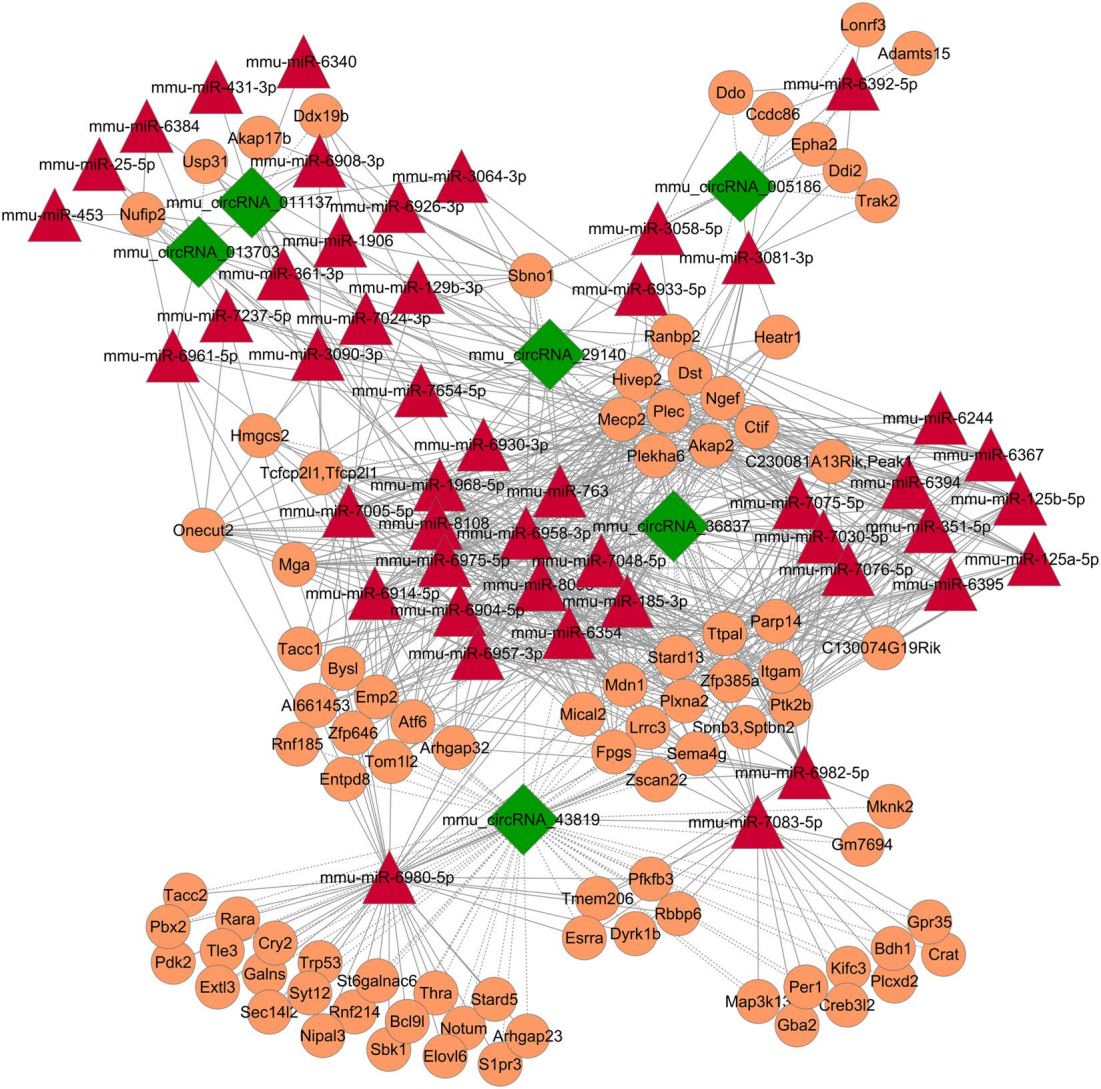
The circRNA-miRNA-mRNA network analysis. The network consists of 6 circRNAs, 47 miRNAs and 90 mRNAs.

We predicted potential interactions among the identified circRNAs, miRNAs and target genes. A ceRNA network of circRNA-miRNA-mRNA interactions, including 6 circRNAs, 47 miRNAs and 90 mRNAs, was constructed by Cytoscape software (Fig. 5). According to the ceRNA network, circRNA, a competing endogenous RNA, increased target gene expression by removing the inhibitory effect of miRNA. 10 predicted miRNAs and mRNAs were selected from the ceRNA network to validate among the three groups by qRT-PCR, respectively. The results indicated that the expression of miR-124-3p, miR-153-3p, miR-210-5p, miR-107-3p, miR-103-3p, miR-205-5p, miR-296-5p, miR-188-3p and miR-698-5p significantly increased and were inversely correlated with their corresponding circRNAs expression (Fig. 4b). Meanwhile, qRT-PCR analysis revealed the expression of Epha2, Arhgap32, Heatr1, Stat3, IL1r1 and Egr1 were significantly different among three groups while only Epha2, Arhgap32, Heatr1 and Egr1 had an identical expression trend with their corresponding circRNAs (Fig. 4c). Based on the verified qRT-PCR data, several circRNA-miRNA-mRNA pathways were constructed, including mmu_circRNA_005186-miR-124-3p-Epha2 and mmu_circRNA_43819-miR-210-5p-Egr1. Since mmu_circRNA_005186 had a higher fold change in qRT-PCR and Epha2 was its linear gene, we therefore chose mmu_circRNA_005186-miR-124-3p-Epha2 pathway for further intensity study.

### mmu_circRNA_005186 acted as a miRNA Sponge for miR-124-3p and regulated the expression of Epha2

**Figure 6:**
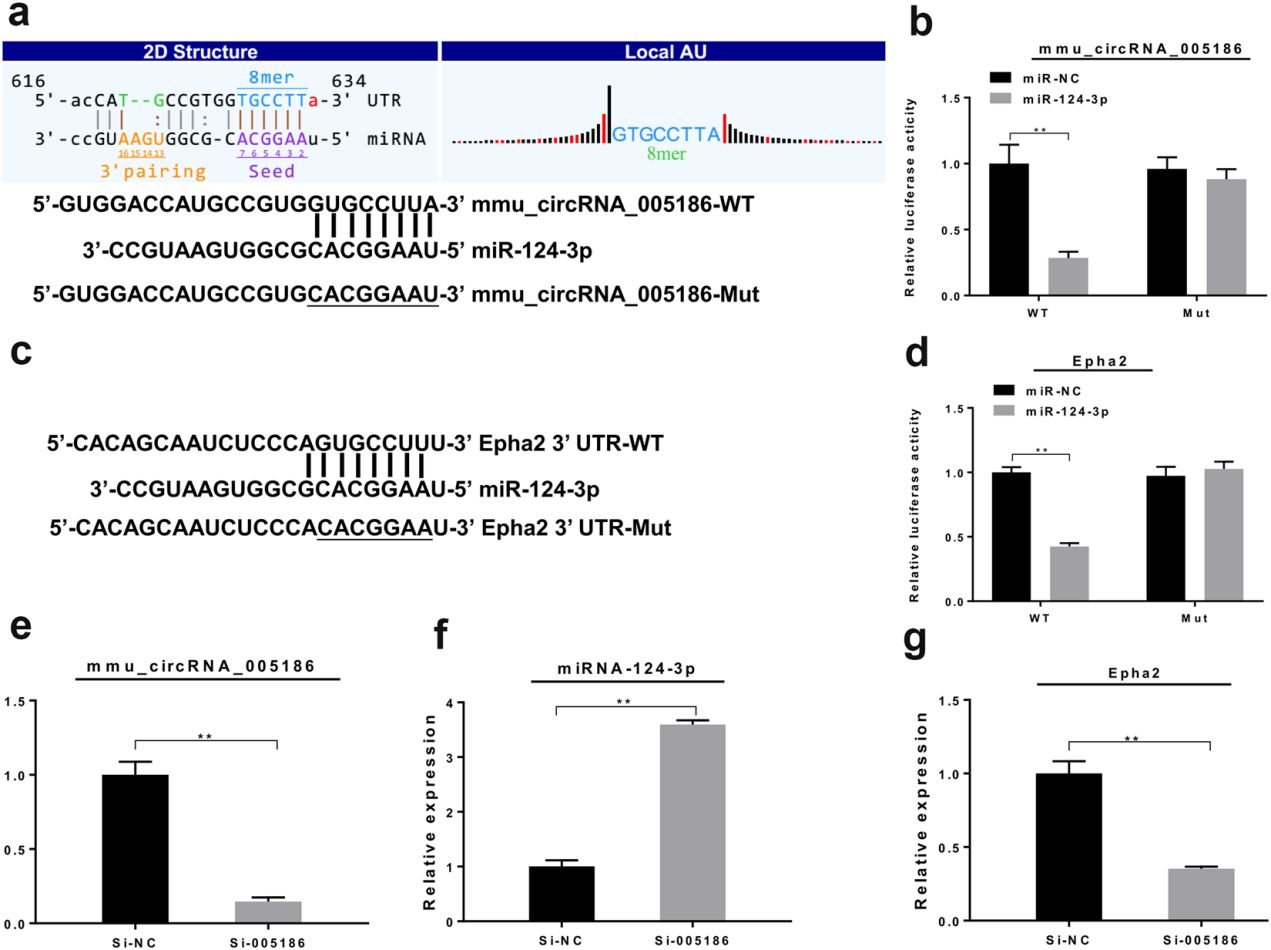
mmu_circRNA_005186 acted as a miRNA Sponge for miR-124-3p and regulated the expression of Epha2. (**a**) Predicted binding site of miR-124-3p in mmu_circRNA_005186 and a diagram for construction of mutated reporter plasmid (mmu_circRNA_005186-Mut). **(b)** Dual-luciferase reporter assay showed that miR-124-3p mimics significantly repressed the luciferase activity of mmu_circRNA_005186-WT but not the mutant one in 293T cells. **(c)** Predicted binding site of miR-124-3p in Epha2 3′-UTR. And a diagram for construction of mutated reporter plasmid (Epha2 3′-UTR-Mut). **(d)** Dual-luciferase reporter assay showed that miR-124-3p mimics significantly repressed the luciferase activity of Epha2 3′-UTR-Mut but not the mutant one in 293T cells. **(e)** After transfection with si-005186, the expression level of mmu_circRNA_005186 was significantly inhibited in RAW264.7 cells. **(f)** The expression level of miR-124-3p was significantly upregulated after mmu_circRNA_005186 silencing in RAW264.7 cells. **(g)** The expression level of Epha2 was significantly inhibited after mmu_circRNA_005186 silencing in RAW264.7 cells. *p < 0.05, **p < 0.01.

To determine whether mmu_circRNA_005186 directly targets miR-124-3p through the predicted binding sites (Fig. 6a), we performed a dual-luciferase reporter assay and found that miR-124-3p mimics significantly reduced the luciferase activity of mmu_circRNA_005186-WT (*p* < 0.01) but not the mutant one in 293T cells (Fig. 6b). Then, we applied the dual-luciferase reporter assay to validate whether miR-124-3p interacts with Epha2 by the predicted binding sites (Fig. 6c). The results indicated that luciferase activity was significantly lower in the miR-124-3p mimic (*p* < 0.01) than the negative control (NC), but this reduction was not observed with the mutant plasmids (Fig. 6d). Furthermore, by qRT-PCR, we found that the expression level of miR-124-3p was significantly upregulated after mmu_circRNA_005186 silencing in RAW264.7 cells (Fig. 6e,f). In addition, the expression level of Epha2 was significantly downregulated (Fig. 6g). Collectively, the above results demonstrated that mmu_circRNA_005186 enhanced Epha2 expression level by acting as a miRNA sponge for miR-124-3p *in vitro*.

### Effect of mmu_circRNA_005186 on LPS-induced inflammation in RAW264.7 cell

**Figure 7:**
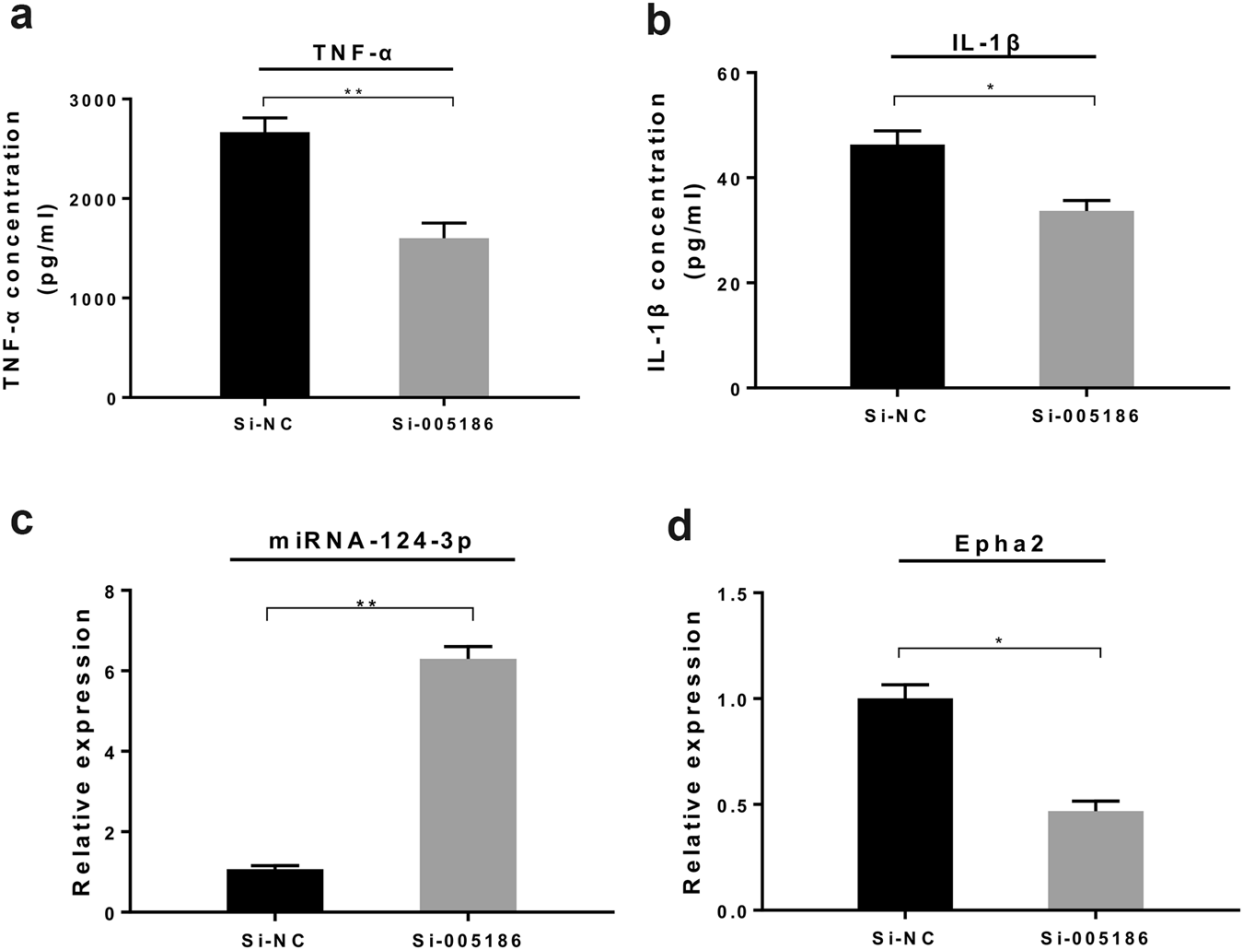
Effect of mmu_circRNA_005186 on LPS-induced inflammation in RAW264.7 cells. **(a**,**b**) ELISA assay demonstrated that the supernatant concentrations of TNF-α and IL-1β released from RAW 264.7 cells were significantly lower in the si-005186 group than the negative control. **(c)** qRT-PCR showed that miR-124-3p was upregulated by 6-fold in the si-005186 group. **(d)** mmu_circRNA_005186 silencing inhibited the expression level of Epha2 in LPS-induced inflammation. *p < 0.05, **p < 0.01.

Given that macrophages play a vital role in hepatic I/R injury, we sought to determine whether mmu_circRNA_005186 affects the LPS-induced inflammation in RAW264.7 cells through the mmu_circRNA_005186-miR-124-3p-Epha2 pathway. The RAW 264.7 cells were transfected with small interfering RNA against mmu_circRNA_005186 (si-005186) or si-circRNA-NC for 48 h and were stimulated by LPS. The supernatant concentrations of TNF-α and IL-1β released from RAW 264.7 cells were significantly lower in the si-005186 group than the negative control (Fig. 7a,b). As expected, qRT-PCR identified that miR-124-3p was upregulated by 6-fold (*p* < 0.01) (Fig. 7c) while the expression level of Epha2 markedly decreased in the si-005186 group (Fig. 7d).

## Discussion

Currently, IPO, an effective method for attenuating hepatic I/R injury^13^, has been used for several years; however, the beneficial mechanism of IPO is still undefined and vague. CircRNAs, a novel type of endogenous noncoding RNA that was recently rediscovered, has gradually been recognized as a molecular biomarker^15^ and as a main mediator in the pathogenesis of tumor growth and metastasis^16^. For example, Hsa_circ_0001649 has been reported as a potential novel biomarker for hepatocellular carcinoma^17^. However, circRNA expression profiles in hepatic I/R injury have not been studied.

In the present study, we used microarray hybridization to detect differential expression profiles and to investigate the biological function of circRNA. A total of 1599 circRNAs were DE among the normal, I/R and IPO groups. A Venn diagram analysis indicated that the majority of the dysregulated circRNAs (51.3% and 58.6%) were sequentially expressed during IPO-induced I/R injury attenuation, which indicated that circRNA plays a vital role in I/R pathogenesis and IPO.

Increasing evidence indicates that circRNA, acts as a competing endogenous RNA or sponge, increases target gene expression by removing the inhibitory effect of miRNA^4,18^. For instance, miR-7a prevents myocardial ischemia-induced cell death by targeting inflammatory and apoptotic genes, however, the circRNA ciRS-7 sponges miR-7a, blocking the anti-apoptotic and cell-protective functions^19^. The heart-related circRNA HRCR is known to prevent cardiac hypertrophy and heart failure by sponging miR-223, which suppresses ARC expression^20^. In the present study, circRNA verification experiments revealed six dysregulated circRNAs following qRT-PCR amplification. To study the potential “housekeeping” functions of these identified circRNAs, a ceRNA network of circRNA-miRNA-mRNA interactions was constructed that includes 6 circRNAs, 47 miRNAs and 90 mRNAs. According to the validation results of selected miRNAs and mRNAs, several ceRNA pathways were constructed. Since the mechanism of circRNA has not been well elucidated, none of the potential circRNA-miRNA-mRNA pathways included in this study have been reported previously. The dual-luciferase analysis validated that miR-124-3p interacts directly with mmu_ circRNA_005186 and Epha2 through the predicted binding sites. The results suggest that mmu_circRNA_005186, serving as a miRNA sponge for miR-124-3p, regulated the expression of Epha2. Furthermore, we explored the mechanism of mmu_circRNA_005186 in LPS-induced RAW264.7 cells that simulated the inflammation in hepatic I/R injury. Through these functional experiments, we demonstrated that mmu_circRNA_005186 silencing attenuated the inflammation and was associated with miR-124-3p upregulation and Epha2 downregulation *in vitro*. Previous studies indicated that miR-124-3p commonly serves an integral roles in cell growth, apoptosis, invasion and metastasis in various cancers^21–23^. In addition, a recent study conducted by Shan Huang indicates that increased miR-124-3p inhibits neuronal inflammation and contributes to neurite outgrowth via their transfer into neurons in a mouse model with traumatic brain injury^24^, suggesting the potential anti-inflammatory role of miR-124-3p. Furthermore, several papers reported that miR-124-3p could be useful molecular marker in brain ischemic stroke^25,26^. Collectively these studies confirm that consistent with our present results, miR-124-3p may inhibit inflammation and attenuate hepatic I/R injury. Epha2 belongs to the membrane-bound tyrosine kinase family, which serves as an oncogene involved in promoting the formation and progression in different types of malignancies^27,28^. In the present study, Epha2 is the linear gene of mmu_circRNA_005186, and this linear gene has been reported to modulate HCC radiosensitivity via p38/mitogen-activated protein kinase-mediated signaling pathways and miR-26b^29^. Ligand-stimulated EphA2 inhibits Akt in PTEN-deficient prostate cancer cells, glioma cells^30^, and other cell types^31,32^ and the activation of mTOR/Akt signaling pathway could protect hepatocytes from hepatic IR injury^33^, suggesting the potential regulating function of Epha2 in hepatic I/R injury. In addition, Saeki *et al*. reported that Epha2 promotes cell adhesion and spreading of monocyte and macrophage cell lines^34^. To the best of our knowledge, platelet and neutrophil adhesion explains the mechanism of hepatic I/R injury^35^. These studies provide novel insights and clues that Epha2 can be applied to study hepatic I/R injury.

In summary, our results show that 1599 circRNAs are dysregulated in hepatic I/R and IPO, and that these circRNAs might play a crucial role in these processes with potential pathophysiological functions. Moreover, we reported for the first time that mmu_circRNA_005186 exerts its function by serving as a miRNA sponge for miR-124-3p, thereby promoting the function of Epha2. Combining the preceding evidence with our functional experiments, modulation of the mmu_circRNA_005186_miR-124-3p_Epha2 pathway may be one of the possible targets and strategy to attenuate hepatic I/R injury.

## Methods and Materials

### Ethics approval

This research protocol was approved by the Committee on the Ethics of Animal Experiments of the Third Xiangya Hospital (No. LLSC (LA) 2016-030) and was conducted according to the Guidance for the Care and Use of Laboratory Animals of the National Institutes of Health.

### Animal model

A total of eighteen 8-week-old male SPF C57BL/6 mice were provided by Hunan SLAC Laboratory Animals (Hunan, China). All mice were housed in a standard room with ad libitum water, rodent food and a 12/12 h light/dark cycle illumination for two weeks. The model of partial (70%) hepatic I/R was used in accordance with previous reports^36^.After an acclimatization period, the eighteen mice were randomly divided into three groups: (1) the normal (N) group, (2) the ischemia/reperfusion (I/R) group and (3) the I/R + ischemic postconditioning (IPO) group. Group N underwent laparotomy without vessel blockage; in the I/R group, liver ischemia was induced for 1 h and reperfusion for 4 h; the IPO group received three intermittent cycles of 5 seconds of ischemia, followed by 5 seconds of reperfusion before the reperfusion phase.

### Cell Culture and Treatment

RAW 264.7 and 293T cells were purchased from the American Type Culture Collection (ATCC, USA) and cultured in DMEM (Gibco, USA) supplemented with 10% fetal bovine serum (HyClone, USA) according to the standard guidelines.

Si-005186 was synthesized by Geneseed Biotech (Guangzhou, China) and the si-005186 sequence is 5′- GATGCCTGCCGAGTTGTTT -3′. RAW 264.7 cells were transfected with si-005186 or si-circRNA-NC using lipofectamine 2000 (Invitrogen, USA) for 48 hours according to the manufacturer’s protocol.

RAW 264.7 cells were seeded at 2 × 10^6^ cells in 1 ml of DMEM in 6-well plates and allowed to adhere for 12 h before stimulation by LPS (100 ng/ml, Sigma, USA). Cells were incubated with LPS for 12 h or 24 h.

### Histological examination of the liver

Hematoxylin-eosin (H&E) staining was conducted to evaluate liver pathological injury. The grade of liver injury was evaluated via Suzuki’s criterion^37^, which indicates the severity of sinusoidal congestion, cytoplasmic vacuolation, and parenchymal cell necrosis.

### Total RNA isolation and quality assessment

Total RNA was extracted from nine frozen mouse liver tissues (three randomly selected samples from each group) and cells using TRIzol (Invitrogen, USA) according to the manufacturer’s instructions. Subsequently, total RNA integrity and gDNA contamination was tested by denaturing agarose gel electrophoresis, and the concentration and quality of the total RNA samples were assessed by a NanoDrop ND-1000 instrument (NanoDrop, USA).

### qRT-PCR

The cDNA synthesis and PCR amplification were carried out by a GeneAmp PCR system 9700 (Applied Biosystems, USA) and ViiA 7 real-time PCR system (Applied Biosystems, USA), respectively. RNA expression was defined as the threshold cycle (Ct). GAPDH was amplified as the internal control for circRNAs and mRNAs and U6 was amplified as the internal control for miRNAs. The relative expression levels were calculated by the double-standard curve method. All primers (Supplementary Table S1) were designed and synthesized by Kangchen Bio-tech (Shanghai, China).

### RNA labelling and circRNA microarray hybridization

RNA labelling and array hybridization were performed according to the manufacturer’s protocol. The enriched circRNAs were amplified and transcribed into fluorescent cRNA. The labeled cRNAs were purified by a RNeasy Mini Kit (Qiagen). The concentration and specific activity of the labelled cRNAs (pmol Cy3/μg cRNA) were measured by a NanoDrop ND-1000 instrument. Each labelled cRNA (1 μg) was fragmented by the addition of 5 μl of 10× blocking agent and 1 μl of 25× fragmentation buffer; then, the mixture was heated at 60 °C for 30 min, and finally, 25 μl of 2× hybridization buffer was added to dilute the labeled cRNA. Hybridization solution (50 μl) was dispensed onto the gasket slide, and this slide was placed onto the circRNA expression microarray slide. The hybridized arrays were washed, fixed and scanned using an Agilent Scanner G2505C.

### CircRNA expression profile data analysis

Agilent Feature Extraction software (version 11.0.1.1) was used to analyse acquired array images. When the data were extracted, quantile normalization and subsequent data processing were conducted using the R software Limma package. CircRNAs that were significantly DE between the two groups were identified through fold change and *p*-values (fold change ≥2.0 and *p*-value < 0.05). The log2 ratio was used for quantile normalization. Hierarchical clustering was conducted to show distinguishable circRNA expression profiles among the samples. Significantly DE circRNAs among the three groups were identified through volcano plot filtering.

### Annotation of linear transcripts and GO and KEGG pathway analysis of host genes

Linear transcripts overlapping with the chromosome location of the circRNA sequence were annotated. CircRNA distribution in the genome was determined by comparing the circRNA with genetic elements. GO analysis was used to investigate three domains of functionality: BP, CC and MF. Fisher’s exact test in the Bioconductor topGO package was used to determine if more overlap existed between the DE list and the GO annotation list than would be expected by chance. The *p*-value produced by the topGO analysis denotes the significance of the GO term enrichment in the DE genes (*p* < 0.05). Pathway analysis was performed to functionally analyze and map genes to KEGG pathways. The *p*-value denotes the significance of the pathway correlated with the conditions (*p* < 0.05).

### Validation of selected circRNAs, miRNAs and mRNAs

Ten circRNAs were selected on the basis of a combination of *p*-value, fold change, raw intensity and type. Ten miRNAs and mRNAs were selected according to the ceRNA network. In addition, further qRT-PCR validation was performed by a SYBR Green PCR kit (Arraystar, USA) in triplicate for each sample.

### Prediction of miRNAs and mRNAs related to circRNAs and construction of the circRNA-miRNA-mRNA Network

To identify predicted circRNA and miRNA interactions, Arraystar’s in-house miRNA target prediction software was applied on the basis of TargetScan and miRanda algorithms. To improve prediction reliability, we set the context + score to less than −0.5. Then, the potential miRNA targets were predicted with miRanda and TargetScan databases, and the ceRNA network of circRNA-miRNA-mRNA was delineated using Cytoscape software.

### Luciferase reporter assay

The sequence of mmu_circRNA_005186 or the 3′-UTR of the Epha2 mRNA, including the seed sequence of the miR-124-3p binding site and a mutated binding site, was cloned into the pmirGLO dual-luciferase vector (Promega, USA).Then, 293T cells were co-transfected with miRNA-124-3p mimics and luciferase vectors using lipofectamine 2000 (Invitrogen, USA). After incubation for 48 h, firefly and Renilla luciferase activity was measured using the DualGlo luciferase assay system (Promega) according to the standard protocol.

### ELISA assay for TNF-α and IL-1β

After treatment with LPS (100 ng/ml) for 24 h, the supernatant concentrations of TNF-α and IL-1β released from RAW 264.7 cells were measured by an automated microplate reader (Heales, China) using commercial ELISA kits according to the instructions of the kits (Abcam, USA).

### Statistical analysis

The microarray data are reported as the means ± SEM. All other statistical data were analyzed and visualized by GraphPad Prism 5.0 and Student’s t-test. The *p-*values were used to evaluate statistical significance (*p* < 0.05).

## Electronic supplementary material

Supplementary Table S1

## Data Availability

The datasets generated during and/or analysed during the current study are available from the corresponding author on reasonable request or GEO database (GSE117524).
