# Supplementary material for: Comprehensive circRNA expression profile during ischemic postconditioning attenuating hepatic ischemia/reperfusion injury
Source: Sci Rep. 2019 Jan 22;9:264. doi: 10.1038/s41598-018-36443-8 (PMC6342922; doi:10.1038/s41598-018-36443-8)
Supplement: Supplementary file 1 — Supplementary Table S1 [file 41598_2018_36443_MOESM1_ESM.pdf]

Comprehensive circRNA expression profile during ischemic postconditioning attenuating hepatic ischemia/reperfusion injury

Pengpeng zhang, Yingzi Ming, Qifa Ye*, Ying Niu*

Table S1 The primers sequence used in this study

ID	Primer	product length (bp)
GAPDH(MOUSE)	F:5’ CACTGAGCAAGAGAGGCCCTAT3’<br>R:5’ GCAGCGAACTTTATTGATGGTATT3’	144
mmu_circRNA_005186	F:5’ GTGCCAGGAAGGCTACGAGA 3’<br>R:5’ TGCGAAGTCCAACAAAACAAC 3’	61
mmu_circRNA_011137	F:5’ CCACGTCTTCTACTAGAGATTGAA 3’<br>R:5’ TCACTTGACTTGAGGAGAGCTG 3’	136
mmu_circRNA_013703	F:5’ CGCCAAAGAACAGAGATACCAG 3’<br>R:5’ TACCGTTTATTTCACCATAGCCT 3’	113
mmu_circRNA_29064	F:5’ CGAAGACCAGAAGAAAATGACACA 3’<br>R:5’ AGCGGTTGATGGCACAGTTG 3’	84
mmu_circRNA_29140	F:5’ ACCAGTTGAAAGGTGCCCCTG 3’<br>R:5’ TTGCTGTCTCCGCTTGAGTCG 3’	132
mmu_circRNA_30491	F:5’ CATGTGGGCAGGACCAAGAG 3’<br>R:5’ GCTGAAGGGGATGCTGTATTCC 3’	98
mmu_circRNA_32403	F:5’ ACTATAAAGCCCAAACAGCAAG 3’<br>R:5’ TTTCAAGGCAAGGCAAGTAAC 3’	103
mmu_circRNA_36837	F:5’ TAAAGCGGCAGAGACAAGTGA 3’<br>R:5’ GGTTAGCATCCTCAGAAAGCTG 3’	130
mmu_circRNA_43819	F:5’ CATTTTTACTCTGGCTCACCC 3’<br>R:5’ CTAGGTCCCACATCCTTGTTC 3’	92
mmu_circRNA_44049	F:5’ GCAGAAACAGAGCCAGCAGAT 3’<br>R:5’ CACTCTTCAGCAGGGAGATGG 3’	148
Epha2	F:5' AGGGAGAAGGATGGTGAGTT 3'<br>R :5’ CTTCCAGCACACGCGAC 3’	184
Arhgap32	F:5' GCAGCGTAATGAAAGTGAGC 3'<br>R :5’ GGTCGGAACAACTTGGAAT 3’	144
Heatr1	F:5' TCAGCTAGTCACCACGCTAA 3'<br>R:5’GAACTCAGTATCTGCCTCTAAAA 3’	139
Stat3	F:5' ACTTCAGACCCGCCAACA 3'<br>R :5’ CCAGCAACCTGACTTTCG 3’	236
IL1r1	F:5' TGTGTGCCCTTATGTGAGTTAT 3'<br>R :5’ TTTACTCCGAAGAAGCTCAC 3’	114
Egr1	F:5' GAGCGAACAACCCTATGAG 3'<br>R :5’ GTCGTTTGGCTGGGATAA 3’	102

Psen2	F:5' CTATCAAGTCTGTGCGTTTCT 3'<br>R :5’ CCCGAGGTAGATGTAGGTGA 3’	254
Hif1a	F:5' GGTGGATATGTCTGGGTTGA 3'<br>R :5’ GATTCTGTTTGTTGAAGGGAG 3’	143
Foxo3	F:5' ACGGCTCACTTTGTCCCA 3'<br>R :5’ GTGCCGGATGGAGTTCTT 3’	109
Ahr	F:5' TGTCATCCATCAGAGCGTAT 3'<br>R :5’ GCTTCGTCCACTCCTTGT 3’	111
U6	F:5’GCTTCGGCAGCACATATACTAAAAT3’<br>R:5’CGCTTCACGAATTTGCGTGTCAT3’	89
mmu-miR-153-3p	GSP:5'GGGGGTTGCATAGTCACAAAA3’<br>R:5’GTGCGTGTCGTGGAGTCG3’	65
mmu-miR-205-5p	GSP:5'CGTCCTTCATTCCACCG3’<br>R:5’CAGTGCGTGTCGTGGAGT3’	64
mmu-miR-103-3p	GSP:5’GGGGAGCAGCATTGTACAGG3’<br>R:5'CAGTGCGTGTCGTGGAGT3'	65
mmu-miR-107-3p	GSP:5’GCAGCAGCATTGTACAGG3’<br>R:5'CAGTGCGTGTCGTGGAGT3'	65
mmu-miR-124-3p	GSP:5’ GGGTAAGGCACGCGGT3’<br>R:5'GTGCGTGTCGTGGAGTCG3’	62
mmu-miR-188-3p	GSP:5’GGGTATCTCCCACATGCAGG3’<br>R:5’GTGCGTGTCGTGGAGTCG3’	65
mmu-miR-221-5p	GSP:5’GGGAACCTGGCATACAATGTAG3’<br>R:5’GTGCGTGTCGTGGAGTCG3’	68
mmu-miR-210-5p	GSP:5’GAAAAGCCACTGCCCACC3’<br>R:5’GTGCGTGTCGTGGAGTCG3’	64
mmu-miR-698-5p	GSP:5’GGGAAAGTGGGTGGGACAG3’<br>R:5’GTGCGTGTCGTGGAGTCG3’	64
mmu-miR-296-5p	GSP:5’GAAGGGCCCCCCCTCA3’<br>R:5'GTGCGTGTCGTGGAGTCG3’	61
